# Numerical testing method and mechanical property evaluation of large particle size asphalt mixture

**DOI:** 10.1371/journal.pone.0316191

**Published:** 2025-01-15

**Authors:** Chengduo Qian, Minghao Mu, Bingchen Zhu, Xinqiang Liu, Zheng Wang, Haisong Bi, Yingjun Jiang

**Affiliations:** 1 Innovation Research Institute of Shandong Hi-Speed Group Ltd, Jinan, Shandong, China; 2 Key Laboratory for Special Area Highway Engineering of Ministry of Education, Chang’an University, Xi’an, Shaanxi, China; Shandong University of Technology, CHINA

## Abstract

The large particle size asphalt mixture with nominal maximum aggregate size 53 mm(LSAM-50) has good technical and economic performance and will become an effective technical way to build a full-thick long-life asphalt pavement with Chinese characteristics. In order to reveal the mechanical properties and influencing factors of LSAM-50 in depth, a numerical test method for the mechanical properties of the large particle size LSAM-50 asphalt mixture was developed, and a reasonable specimen size for LSAM-50 performance test was proposed by combining the numerical test and the indoor test. The results show that: LSAM-50 numerical test conditions are the calculation time step 10^−3^ s/step, the loading rate is 2 mm/min (uniaxial compression numerical test) and 50 mm/min (splitting numerical test) when LSAM-50 numerical experiment calculation rate and numerical experiment accuracy are better; after the size of the specimen reaches 200×160mm, the influence of the size effect is eliminated. The reasonable specimen size of LSAM-50 is Φ200mm×h160mm; the LSAM-50 numerical test method and the indoor test have low error Less than 10%.

## 1. Introduction

In China, over 90% of high-grade highways use semi-rigid base asphalt pavements. However, the issue of cracking in these pavements has been unresolved [1], making the development of long-lasting asphalt pavements a significant research topic in road engineering. Some countries have turned to full-depth asphalt pavements to avoid cracking at the bottom of the asphalt layer and structural rutting [2–7]. Asphalt mixtures with large aggregate sizes have gained attention globally for their high strength, rut resistance, and durability, showing considerable advantages in delaying crack propagation [8–11]. Moreover, these mixtures require less asphalt and allow for the construction of thicker layers in a single pass. Most studies have focused on asphalt mixtures with a maximum aggregate size of ≤37.5mm. Therefore, our research group has developed a large size asphalt mixture with a maximum aggregate size of 53mm (LSAM-50). LSAM-50’s strength, rut resistance, and cost-effectiveness potentially make it a suitable material for flexible base asphalt pavements. Given that the maximum aggregate size of LSAM-50 exceeds 53 mm, existing test methods and conditions may no longer be applicable to LSAM-50 mixtures. Considering the complex environment and high loads faced by asphalt mixtures in actual applications, it is essential to develop numerical test methods for LSAM-50 to more thoroughly understand and optimize its mechanical behavior and performance characteristics.

Numerical simulation, as an effective research tool, has gained extensive application in material science and engineering technology. This is particularly true in the study of asphalt mixtures, where considering the volume and cost of traditional testing methods, numerical simulation offers a more efficient and viable research alternative. In exploring the micromechanical properties of asphalt mixtures, significant achievements have been made using the finite element method based on continuum mechanics [12–15]. However, asphalt mixtures display non-continuous and discrete characteristics in the distribution of voids and aggregate particles. The finite element method is unable to capture the inter-particle interactions and highly non-linear mechanical behaviors of asphalt mixtures. As a result, many researchers have turned to the discrete element method to study the micromechanical behavior of asphalt mixtures, thereby revealing micromechanisms that are unattainable through indoor experiments [16–20].

Based on this, this paper conducts research on numerical testing methods for large aggregate LSAM-50 asphalt mixtures and reasonable specimen dimensions. Using the Discrete Element Method (DEM), a numerical testing method for LSAM-50 is proposed, including model construction, simulation of testing processes, and optimization of testing conditions. The reliability of numerical testing is verified through laboratory experiments. This research also explores suitable specimen dimensions for LSAM-50 and provides a preliminary investigation into the performance of LSAM-50, serving as a theoretical basis for the promotion of LSAM-50 mixtures and the development of long-lasting asphalt pavements.

## 2. LSAM-50 raw materials and gradation

### 2.1 Raw materials

#### 2.1.1 Asphalt

The asphalt is Zhenhai heavy traffic 70# road petroleum asphalt, and its technical properties are shown in Table 1.

**Table 1:** Technical properties of asphalt.

Items		Tested results	Standard value
Penetration (25°C, 100 g, 5 s) (0.1 mm)		68	60~80
Ductility (5 cm/min, 15°C) (cm)		>100	≥100
Softening point (°C)		47.3	≥46
Penetration index (PI)		-0.315	-1.5~+1.0
Flashpoint (°C)		290	≥260
Solubility in trichloroethylene (%)		99.6	≥99.5
Rolling Thin Film Oven Test (163°C,5h)	Quality loss (%)	0.03	≤±0.8
	Ductility (10°C) (cm)	7	≥6
	Penetration ratio (25°C) (%)	63	≥61

#### 2.1.2 Aggregate

The coarse and fine aggregates are both limestone, and their technical properties are shown in Table 2.

**Table 2:** Technical properties of aggregate.

Aggregate	Items	Tested results	Standard value
Coarse aggregate	Crushed value (%)	19.3	≤26
	Los Angeles Abrasion Value (%)	19.8	≤28
	Flaky particle content (%)	8.1	≤15
	Apparent relative density (g/cm^3^)	2.781	≥2.6
Fine aggregate	Methylene blue value (g/kg)	3.7	≤25
	Sturdiness (%)	6.9	≤12
	Apparent relative density (g/cm^3^)	2.719	≥2.5

### 2.2 LSAM-50 gradation

This paper has selected four different aggregate gradations, detailed in Table 3.

**Table 3:** Aggregate gradation of LSAM-50.

Gradation	Percentage by mass passing through the sieve sizes (mm)											
	63	53	37.5	19	9.5	4.75	2.36	1.18	0.6	0.3	0.15	0.075
JP50-1	100	100	67	45	35	30	25	17.5	13	9.5	6.5	3
JP50-2	100	100	65	45	35	30	25	17.5	13	9.5	6.5	3
JP50-3	100	100	67	49	35	30	25	17.5	13	9.5	6.5	3
JP50-4	100	100	70	55	35	30	25	17.5	13	9.5	6.5	3

### 2.3 Performance of LSAM-50 mixture

The optimal asphalt-aggregate ratio for the LSAM-50 mixture is 2.8%. The volumetric parameters and the road performance of the LSAM-50 mixture at this optimal asphalt-aggregate ratio are presented in Table 4.

**Table 4:** Volumetric parameters and road performance of LSAM-50 mixture.

Indicator	Bulk Density (g/cm^3^)	Void Ratio(%)	Aggregate Void Ratio (%)	Asphalt Saturation(%)	Dynamic Stability (time/mm)	Bending Strain(μξ)	SCB Strength(MPa)	Residual SCB Strength(%)
value	2.548	3.7	7.6	50.5	14021	3357	10.71	90.4

## 3. Numerical test method for LSAM-50

### 3.1 Discrete element method and the basic conditions of the numerical model

The Discrete Element Method (DEM) is a widely employed numerical technique for investigating the behavior of granular materials. By establishing a numerical model that simulates laboratory tests, the method allows for the interaction forces between particles and between particles and walls to be transmitted through contact points. Motion equations are established for each particle and solved to understand the overall deformation and stress distribution. DEM is also a means to investigate the micro-properties of materials and is often used to describe failure processes and to establish macro-micro relationships, among other applications. The effectiveness of DEM in the study of asphalt mixtures has been validated.

In numerical experiments, asphalt mixtures are divided into asphalt mortar (asphalt, mineral powder, and fine aggregates smaller than 1.18mm) and coarse aggregates (aggregates larger than 1.18mm). Theoretically, the particle size distribution, bulk density, and contact state of the numerical model are consistent with those of the laboratory-molded specimens.

DEM utilizes contact constitutive relationships to describe the force-bearing state of the particles. In this paper, different contact models are used to represent different interactions: the linear contact stiffness model for coarse aggregate to coarse aggregate, the parallel bonding model for coarse aggregate to asphalt mortar, and the same parallel bonding model for asphalt mortar to asphalt mortar contacts.

### 3.2 Construction of numerical model

#### 3.2.1 Asphalt mixture simulation

The gradation of asphalt mixtures is usually determined by the mass ratio of aggregates of different particle sizes to the total mass of the mineral material. In PFC 2D, aggregates, mortar, and trial molds are all presented in two-dimensional form. During the model construction process, the aggregate particles are in the form of discs. The default thickness of two-dimensional rigid discs in discrete element software is 1. The grading is achieved in the software by converting the mass ratio of aggregates of different specifications into the area ratio of rigid discs.

If the specimen has a diameter d, a height h, and there are k different specifications of aggregates, and if the standard density of the asphalt mixture specimen is ρ, then the total mass of the specimen can be calculated using Eq(1):

$$M=\rho \times \frac{\pi d^{2}}{4}\times h$$ (1)

In PFC 2D, the mass of the specimen transformed into a two-dimensional form m, is as follows:

m=ρ×S=ρ×D×h (2)

In the equation, S represents the area of the specimen, where *S* = *D*×*h*, cm^2^.

The mass of each aggregate size is calculated using the cumulative passing rate from sieve analysis, as shown in Eq (3), and the area is calculated using Eq (4).


$$m_{i}=\rho \times (1-VV)\times D\times h\times a$$ (3)

$$S_{i}=\frac{m_{i}}{\rho_{i}}$$ (4)

In the formula: m_i_ is the total mass of the i-th aggregate size, g; VV is the void ratio of the specimen, %; a_i_ is the cumulative passing rate of the i-th aggregate size from the sieve analysis; S_i_ is the total area of the rigid discs in two dimensions representing the i-th aggregate size, cm^2^; ρ_i_ is the apparent density of the i-th aggregate size, g/cm^3^.

#### 3.2.2 Generation of aggregate particles

**Fig 1:**
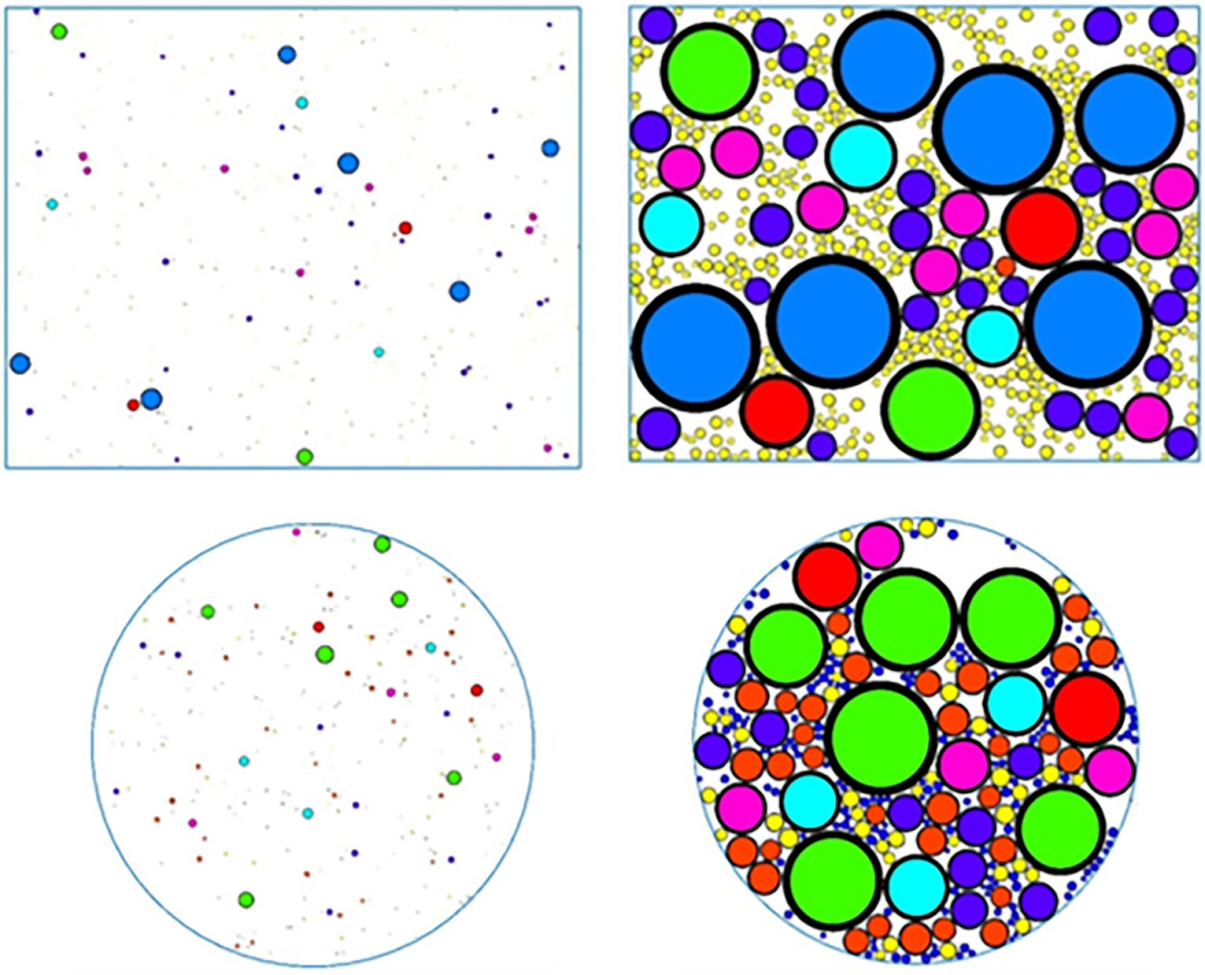
Particle simulation generation process: (a) Uniaxial compression test particle enlargement before (b) Uniaxial compression test particle enlargement after (c) Splitting Test particle enlargement before (d) Splitting test particle enlargement after.

To prevent the generated particles from escaping beyond the wall, a gradual expansion method is used to generate the particles. After determining the number of particles, they are first scaled up by ten times. Once the required number of particles is reached, they are gradually enlarged until they match the original size before scaling. The process of particle generation is shown in Fig 1.

### 3.3 Numerical experimentation process simulation

#### 3.3.1 Uniaxial compression numerical test

The implementation of the uniaxial compression numerical test for the LSAM-50 includes the creation of the mold, the test base, and the penetrating indenter. A cylinder with a diameter of Φ200mm and a height of h160mm is used. In the software, a closed rectangular wall with a height of 160mm and a width of 200mm is generated to simulate the mold. A rectangular wall wider than the mold, coinciding with the bottom surface of the mold, simulates the test base. A closed rectangular wall, slightly wider than the mold, is generated above the mold to simulate the indenter in the uniaxial compression test.

**Fig 2:**
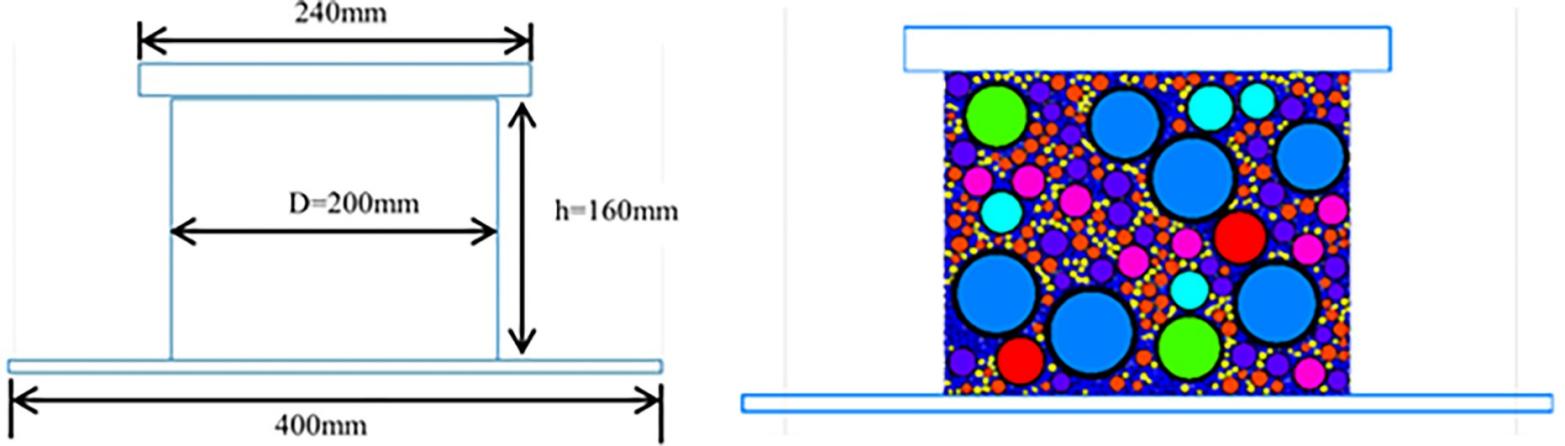
Uniaxial compression numerical test.

After the creation of the LSAM-50 numerical specimen, the mold, test base, and penetrating indenter, the rectangular mold wall is deleted to simulate the demolding process. The penetrating indenter is moved towards the LSAM-50 specimen at a fixed rate of 2mm/min. The uniaxial compression numerical test for the coarse aggregate asphalt mixture is shown in Fig 2. The ’History’ command is used to monitor the force on the contact surface from the moment the indenter comes into contact with the specimen, recording every 50 time steps.

#### 3.3.2 Splitting numerical test

**Fig 3:**
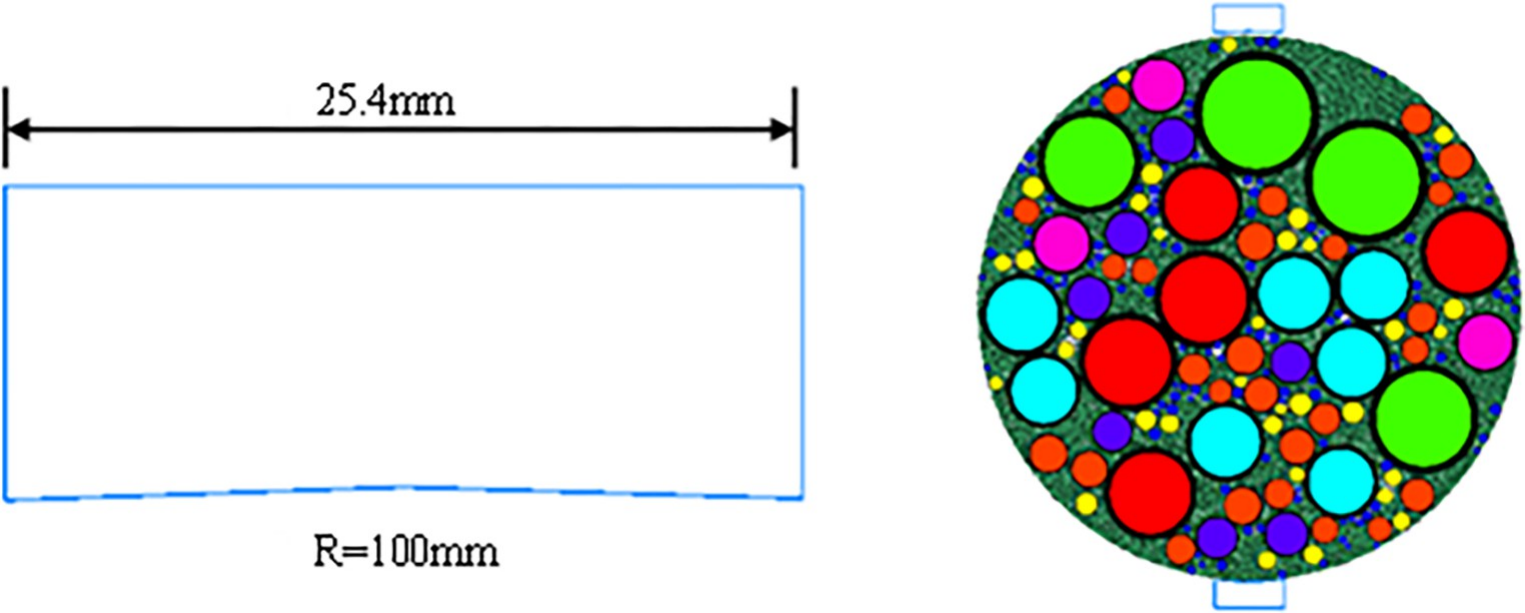
Splitting numerical test: (a) S plit strip (b) test process.

The implementation of the splitting numerical test for the LSAM-50 mixture also involves the generation of molds and splitting strips. The mold is a closed spherical wall with a diameter of 100mm, while the splitting strip has a width of w and a curved surface radius of 25.4mm. The loading rate of the splitting experiment is 50 mm/min. These are created using the Geometry command, as shown in Fig 3(A). After generating the LSAM-50 mixture numerical specimens, molds, and splitting strips, the spherical mold wall is deleted to simulate demolding. The upper splitting strip is then moved downward at a velocity of v2. Upon contact between the upper splitting strip and the numerical specimen, the contact force and displacement between the strip and the LSAM-50 numerical specimen are recorded. The numerical splitting test is depicted in Fig 3(B).

### 3.4 Calibration of numerical model parameters for LSAM-50 mixture

#### 3.4.1 Model parameter sensitivity analysis

**Fig 4:**
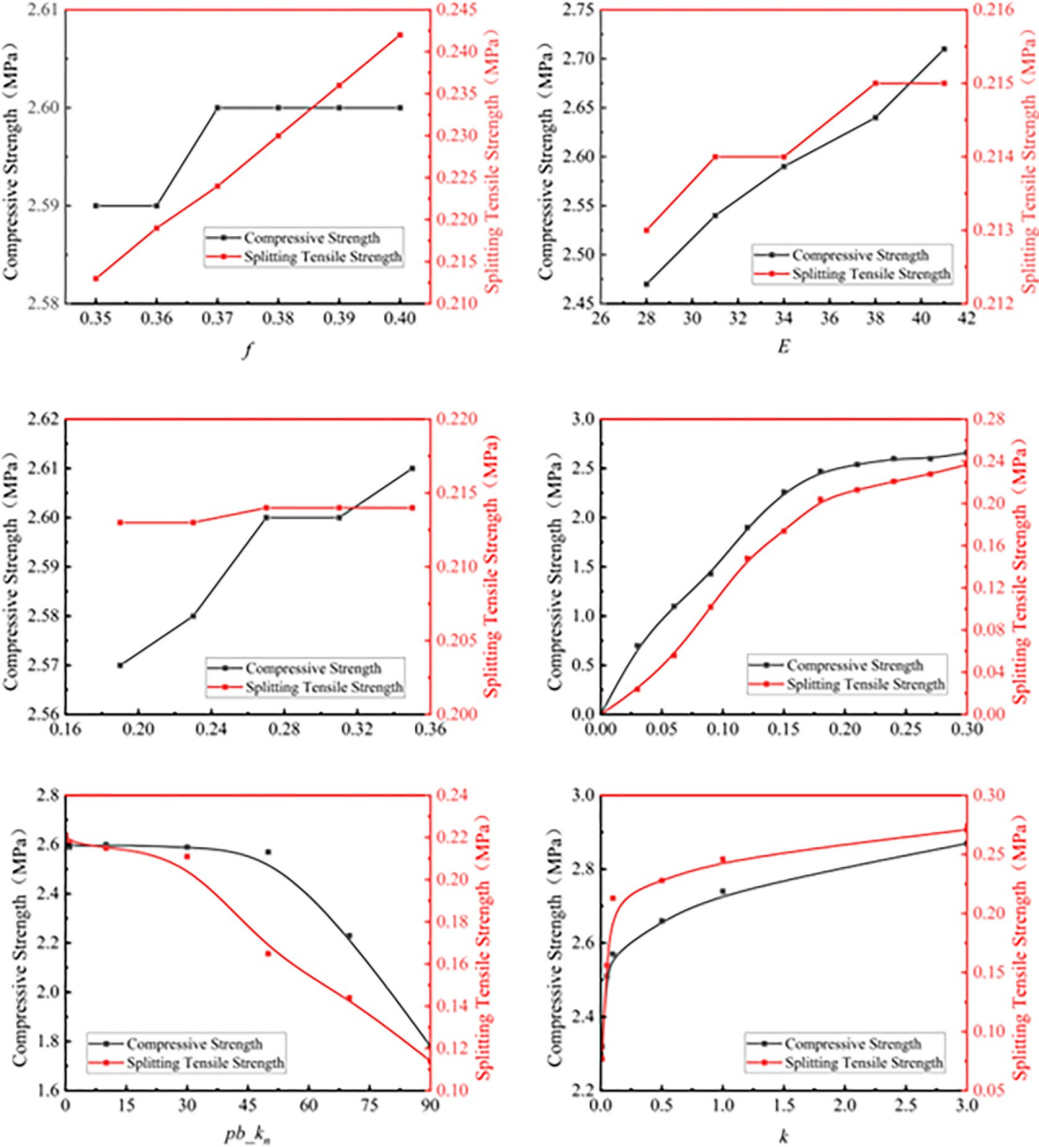
Mechanical strength of LSAM-50 mixture under different model parameters: (a) f (b) E (c) v (d) pb_ten (e) pb_kn (f) k.

The commonly used basic friction coefficient f for parent rock ranges between 0.30 and 0.55. In this paper, as the aggregate is limestone, to study the impact of f on the uniaxial compression numerical test and splitting numerical test results of the LSAM-50 mixture, f is proposed to be set between 0.35 and 0.40, with a step size of 0.01. The Young’s modulus E of limestone typically falls within the range of 28 to 41 GPa. It is advisable to set it within this range. The Poisson’s ratio v is proposed to be between 0.19 and 0.35. The parallel bond strength pb ten is proposed to range from 0 to 0.3 MPa, and the parallel bond stiffness pb kn from 0.1 to 90 GPa. The contact stiffness k of the asphalt mortar is suggested to be between 0.01 and 3 GPa. The mechanical strengths of the LSAM-50 mixture under different model parameters are presented in Fig 4. The specific parameters during the test are shown in Tables 5–10.

**Table 5:** Other model parameters when study f.

Indicator	kn (GPa)	ks (GPa)	v	krat	pb_ten (MPa)	pb_coh (MPa)	pb_kn (GPa)	pb_ks (GPa)	pb_k (GPa)
value	34	13.4	0.27	2.54	0.2	0.2	30	30	0.1

**Table 6:** Other model parameters when study E.

Indicator	f	v	krat	pb_ten (MPa)	pb_coh (MPa)	pb_kn (GPa)	pb_ks (GPa)	pb_k (GPa)
value	0.37	0.27	2.54	0.2	0.2	30	30	0.1

**Table 7:** Other model parameters when study v.

Indicator	f	kn (GPa)	pb_ten (MPa)	pb_coh (MPa)	pb_kn (GPa)	pb_ks (GPa)	pb_k (GPa)	kn (GPa)
value	0.37	34	0.2	0.2	30	30	0.1	34

**Table 8:** Other model parameters when study pb_ten.

Indicator	f	kn (GPa)	ks (GPa)	v	krat	pb_kn (GPa)	pb_ks (GPa)	pb_k (GPa)
value	0.37	34	13.4	0.27	2.54	30	30	0.1

**Table 9:** Other model parameters when study pb_kn.

Indicator	f	kn (GPa)	ks (GPa)	v	krat	pb_ten (MPa)	pb_coh (MPa)	pb_k (GPa)
value	0.37	34	13.4	0.27	2.54	0.21	0.21	0.1

**Table 10:** Other model parameters when study k.

Indicator	f	kn (GPa)	ks (GPa)	v	krat	pb_ten (MPa)	pb_coh (MPa)	pb_kn (GPa)	pb_ks (GPa)
value	0.37	34	13.4	0.27	2.54	0.21	0.21	34	34

As shown in Fig 4(A), with the increase of the friction coefficient f, the compressive strength of the LSAM-50 mixture numerical specimens changes slightly, with an increase of only 1.5%. However, the effect on splitting strength is more pronounced. Therefore, the median value of the limestone friction coefficient (0.38) is selected as the model parameter. Fig 4(B) indicates that for every 1 GPa increase in Young’s modulus E, the compressive strength increases by 0.18 MPa, a relative increase of 0.7%, which has a minor impact on the compressive strength of coarse aggregate asphalt mixture numerical specimens. The variation in splitting strength with Young’s modulus is also minimal, leading to the selection of the median value of E (33 GPa).

From Fig 4(C), it is evident that as the Poisson’s ratio v increases, the compressive and splitting strengths of the asphalt mixture numerical specimens remain almost unchanged, with variations within 1%, indicating minimal impact on the mechanical strength of the specimens. The median value of 0.27 is chosen for the Poisson’s ratio. Fig 4(D) shows that both the compressive and splitting strengths of the LSAM-50 asphalt mixture numerical specimens increase with the increase of the parallel bond normal strength pb ten, and the mechanical strength tends to stabilize when pb ten exceeds 0.2 MPa. Thus, an initial choice of 0.2 MPa is made for pb ten.

According to Fig 4(E), when the parallel bond stiffness pb kn varies between 0.1 and 50 GPa, the compressive strength of LSAM-50 numerical specimens remains relatively constant. However, when pb kn exceeds 50 GPa, a noticeable decline in compressive strength is observed. This decrease is attributed to the instability in the simulation process as the stiffness of the particle contact wall becomes comparable to the increased parallel bond stiffness. The splitting strength remains stable within the range of 0.1 to 30 GPa, leading to the initial selection of 30 GPa for pb kn. Finally, Fig 4(F) indicates that when the mortar contact stiffness k is between 0.01 and 0.1 GPa, the increase in compressive strength of the LSAM-50 mixture numerical specimens is about 10%, and the increase in splitting strength is about 50%. Beyond 0.1 GPa, the asphalt mortar contact stiffness has a negligible impact on the simulation results of the LSAM-50 numerical test. This occurs because, with lower stiffness of the asphalt mortar, the bond strength provided by the parallel bond model in the LSAM-50 mixture numerical specimens is insufficient to maintain stability. Once the asphalt mortar contact stiffness reaches a certain value, the specimens have enough bond strength to remain stable, and the strength gradually increases with the increase in asphalt-aggregate ratio, until it reaches an excess of asphalt at the optimal ratio, beyond which compressive and splitting strengths do not increase. Therefore, an initial value of 0.1 GPa is chosen for the mortar contact stiffness k.

#### 3.4.2 Model parameter calibration

*(1) Preliminary setting of model parameters*. In numerical experiments, the model parameters for normal stiffness kn, shear stiffness ks, and aggregate stiffness ratio krat are calculated according to Eqs (5), (8), and (9), respectively. Based on the preliminarily selected model parameters, the initial model parameters as shown in Table 11 are obtained.


$$k_{n}=\frac{E\times A}{L}=E\times t$$ (5)

$$k_{s}=G\times L$$ (6)

E=2×G×(1+v) (7)

$$k_{s}=\frac{k_{n}}{2(1+v)}=\frac{Et}{2(1+v)}$$ (8)

$$k_{rat}=\frac{k_{n}}{k_{s}}=2+2v$$ (9)

In the formula: ks is the tangential stiffness of the particles, measured in N/m;t is the thickness of the particles expressed in 2D form, which is defaulted to 1 in the software;G is the shear modulus of the aggregate, measured in GPa;v is the Poisson’s ratio of the aggregate.

**Table 11:** Initial model parameters.

Parameter Type	*f*	*k*_*n*_ (GPa)	*k*_*s*_ (GPa)	*V*	*krat*	*pb_ten* (MPa)	*pb_k*_*n*_ (GPa)	*pb_k* (GPa)
Parameter Value	0.38	33	13.0	0.3	2.60	0.2	33	0.1

*(2) Adjustment and determination of model parameters*. The cylindrical specimens, sized Φ200 mm×h160 mm, were subjected to uniaxial compression and splitting tests. The preliminary simulation values and errors for the four different gradations are presented in Table 12. As indicated by Table 12, the initial model parameter simulations resulted in values higher than the experimental results, with the error in splitting strength being greater than that in compressive strength. Based on the sensitivity of the model parameters, adjustments were made by reducing the friction coefficient to 0.35 and lowering the parallel bond normal strength pb ten to 0.18 MPa. The simulation values and errors for the four gradations after these adjustments are shown in Table 13. From Table 13. it can be seen that the adjusted model parameters closely approximate the actual measured values, with errors within 5%. In summary, the suitable model parameters for constructing the LSAM-50 numerical experiment model are as shown in Table 14.

**Table 12:** The simulated values and errors of different gradations under the initial model parameters.

Gradation	JP50-1	JP50-2	JP50-3	JP50-4
Compressive Strength (MPa)	2.73	2.85	2.89	2.95
Splitting Tensile Strength (MPa)	0.230	0.232	0.236	0.239
Error in Compressive Strength (|%|)	5.41	6.34	6.25	6.88
Error in Splitting Tensile Strength (|%|)	7.48	7.41	7.76	7.66

**Table 13:** The simulated values and errors of different gradations under the adjusted model parameters.

Gradation	JP50-1	JP50-2	JP50-3	JP50-4
Compressive Strength (MPa)	2.66	2.77	2.82	2.88
Splitting Tensile Strength (MPa)	0.207	0.21	0.216	0.219
Error in Compressive Strength (|%|)	2.70	3.36	3.68	4.35
Error in Splitting Tensile Strength (|%|)	3.27	2.78	1.37	1.35

**Table 14:** Final model parameters.

Parameter Type	*f*	*k*_*n*_ (GPa)	*k*_*s*_ (GPa)	*v*	*krat*	*pb_ten* (MPa)	*pb_k*_*n*_ (GPa)	*k* (GPa)
Parameter Value	0.35	33	13.0	0.3	2.60	0.18	30	0.5

## 4. Numerical test conditions for LSAM-50 mixture

Experimental conditions influence the outcomes of numerical experiments, primarily encompassing factors such as the computational time step (TS), loading rate (LV), and specimen size (SS). This study investigates the impact of these experimental conditions on the results of uniaxial compression and splitting tests for the LSAM-50 mixture. Based on this investigation, the study proposes suitable numerical test conditions for the LSAM-50 mixture.

### 4.1 Computational time step

The computational time step (Time step) reflects the relationship between simulation time and real time. When the time step value is large, the software runs more efficiently, but it might skip over actual peak values, leading to significant deviations between simulation results and indoor experimental data. A smaller time step ensures the accuracy of contact judgment but significantly reduces computational efficiency, and low-specification graphic workstations may not be able to run the software effectively.

In numerical experiments, time steps are typically set at 10^−2^ s/step, 10^−3^ s/step, or 10^−4^ s/step. At a time step of 10^−2^ s/step, numerical specimens can be generated quickly, but during the loading process, there are significant fluctuations in compressive and splitting strengths with changes in time steps. At a time step of 10^−3^ s/step, changes in compressive and splitting strengths with time steps are more stable. However, at a time step of 10^−4^ s/step, the efficiency of generating numerical specimens drops drastically, and while the changes in compressive and splitting strengths are stable, the decreased computational efficiency is not conducive to subsequent numerical simulation studies.

For this study, a computational time step of 10^−3^ s/step is used for the LSAM-50 mixture numerical experiments.

### 4.2 Loading rate

For uniaxial compression tests, the American Society for Testing and Materials (ASTM) standard ASTM D 1074 specifies a loading rate of 0.05 mm/min, whereas the American Association of State Highway and Transportation Officials (AASHTO) standard AASHTO T 167 recommends a loading rate between 1.3 and 5 mm/min. In contrast, Chinese standards require a loading rate of 2 mm/min. For splitting tests, most standards commonly suggest a loading rate of around 50 mm/min.

**Fig 5:**
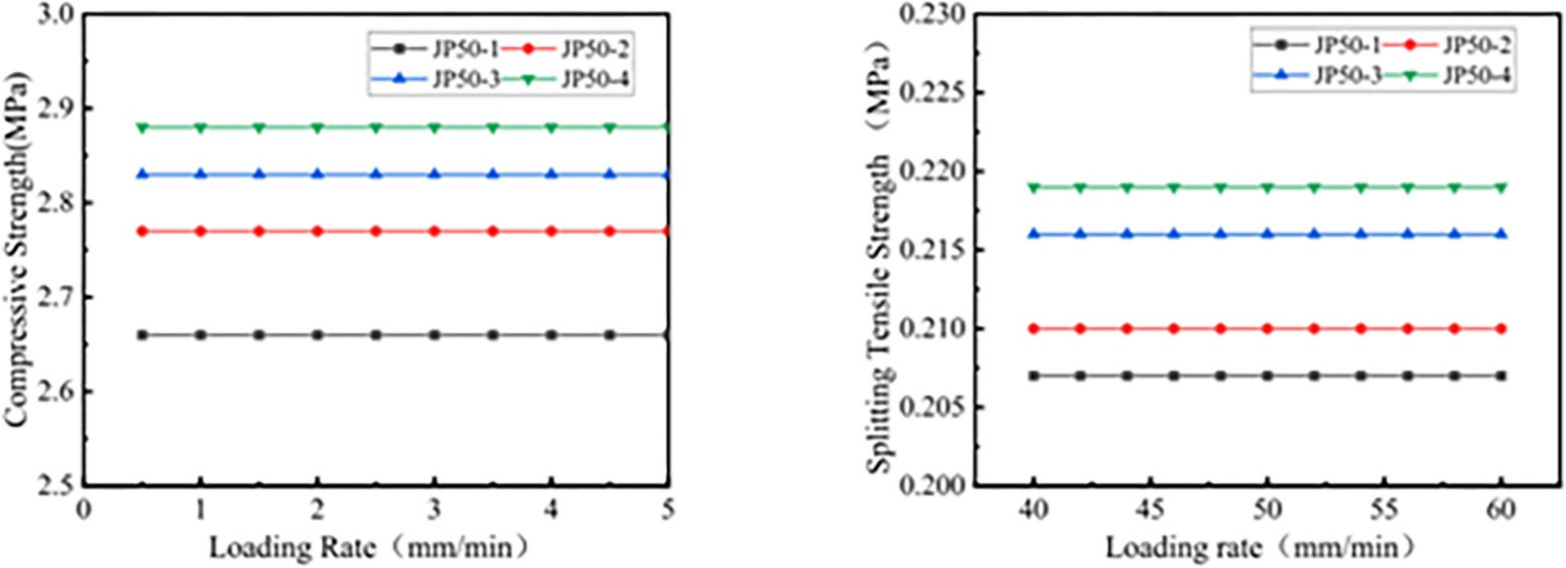
Mechanical strength~loading rate curve: (a) Compressive strength, (b) Splitting strength.

In this study, uniaxial compression numerical tests on different asphalt mixtures were conducted at loading rates ranging from 0.5 to 5 mm/min, with increments of 0.5 mm/min, and at rates between 40 and 60 mm/min, with increments of 2 mm/min. The impact of different loading rates on the simulation results is illustrated in Fig 5.

As seen from Fig 5, for the four types of LSAM-50 mixtures, both compressive and splitting strengths remain unaffected by changes in the loading rate. Therefore, in line with the requirements of Chinese standards, the loading rate for the uniaxial compression numerical test of the LSAM-50 mixture is set at 2 mm/min, and the loading rate for the splitting numerical test is set at 50 mm/min.

## 5. Research on the specimen size of LSAM-50 mixture

### 5.1 Impact of specimen size on mechanical strength

Due to the large nominal maximum particle size of 53 mm in the LSAM-50 mixture, which is significantly larger than that of traditional asphalt mixtures, the traditional specimen sizes may no longer be applicable for the LSAM-50 mixture due to size effects. To mitigate the impact of these size effects, and in line with previous studies, it is identified that the specimen diameter and height should range between 201.4–386.9 mm and 125.9–241.8 mm, respectively. Numerical uniaxial compression and splitting tests were conducted on five different sizes of LSAM-50 mixture specimens (with diameters × heights of 180×160, 200×140, 200×160, 200×180, and 220×160 mm), to study the influence of specimen size on the results of numerical tests and to propose a reasonable specimen size. The results of these numerical tests are presented in Table 15.

**Table 15:** Effect on the mechanical strength of the specimen size LSAM-50 mixture.

Specimen Size/mm	Test Indicators	Mechanical Strength of LSAM-50 Mixture for the Following Gradations / MPa			
		JP50-1	JP50-2	JP50-3	JP50-4
180×160	Compressive Strength	2.82	2.91	3.07	3.13
	Splitting Tensile Strength	0.218	0.225	0.229	0.231
200×140	Compressive Strength	2.75	2.89	3.02	3.07
	Splitting Tensile Strength	0.237	0.242	0.248	0.251
200×160	Compressive Strength	2.66	2.77	2.82	2.88
	Splitting Tensile Strength	0.207	0.211	0.217	0.219
200×180	Compressive Strength	2.61	2.68	2.73	2.79
	Splitting Tensile Strength	0.184	0.188	0.193	0.195
220×160	Compressive Strength	2.57	2.69	2.73	2.80
	Splitting Tensile Strength	0.189	0.194	0.201	0.205

**Fig 6:**
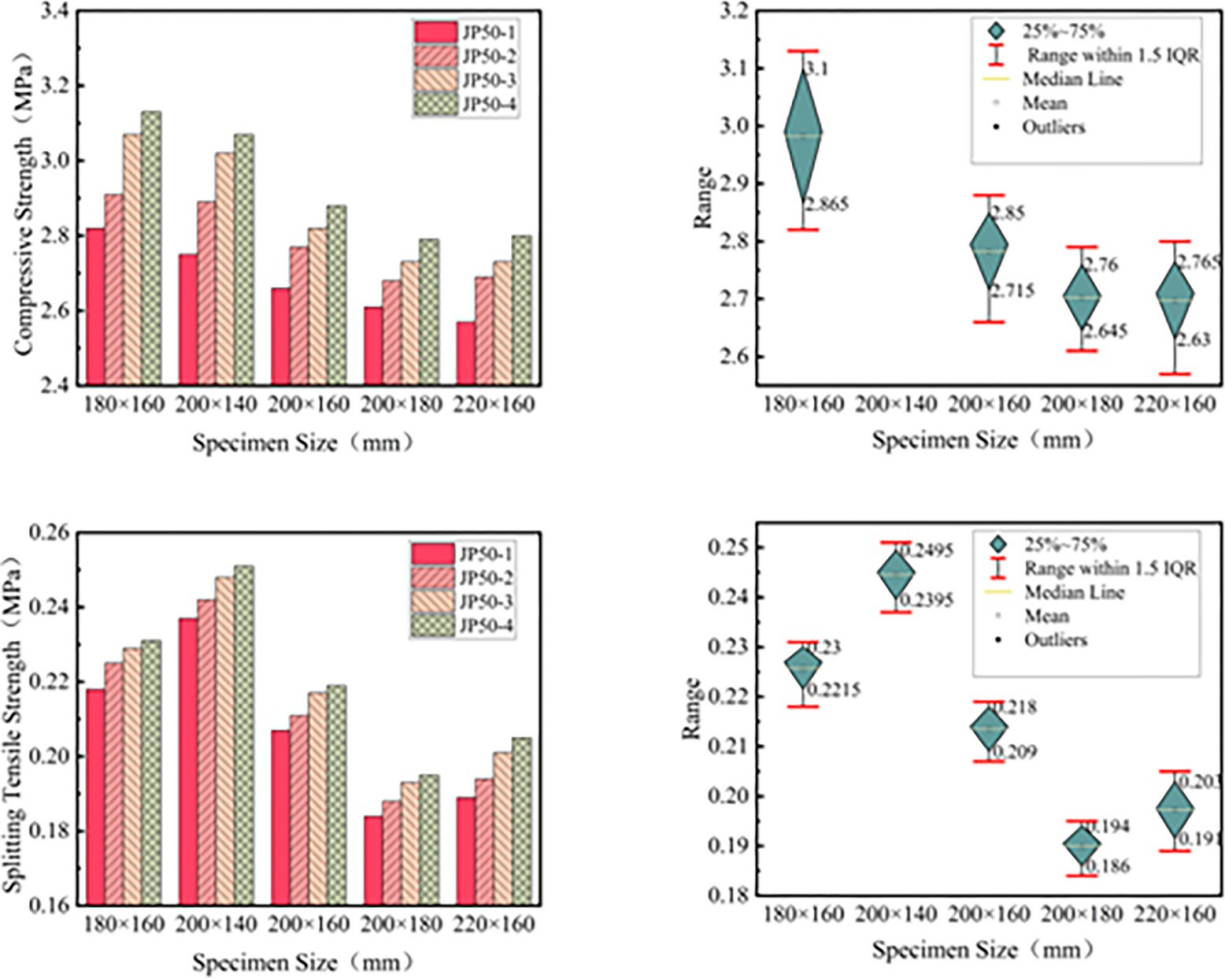
Specimen size ~ mechanical strength relationship diagram: (a) compressive strength relationship diagram, (b) compressive strength box diagram, (c) split strength relationship diagram, (d) split strength box diagram.

Table 9 reveals that specimen size significantly impacts the mechanical strength of the LSAM-50 mixture. As the size of the specimen increases, the mechanical strength of the LSAM-50 mixture gradually decreases. However, after the specimen size reaches a certain threshold, the mechanical strength of the mixture begins to stabilize. To further investigate the effect of size, a graph depicting the relationship between the size of the LSAM-50 mixture specimens and their mechanical strength was created, as shown in Fig 6.

Fig 6 indicates that both the compressive strength and splitting tensile strength of the LSAM-50 mixture decrease with an increase in specimen size. However, the rate of strength reduction diminishes significantly as the specimen size increases. When the specimen size reaches 200×160 mm, further increasing the size results in only a 5% and 8% decrease in compressive strength and splitting tensile strength, respectively. This suggests that a specimen size of 200×160 mm effectively mitigates the impact of size effects. While further increasing the specimen size might enhance the accuracy of indoor tests, it also increases the complexity and difficulty of conducting these tests.

### 5.2 Impact of specimen size on displacement vector field

**Fig 7:**
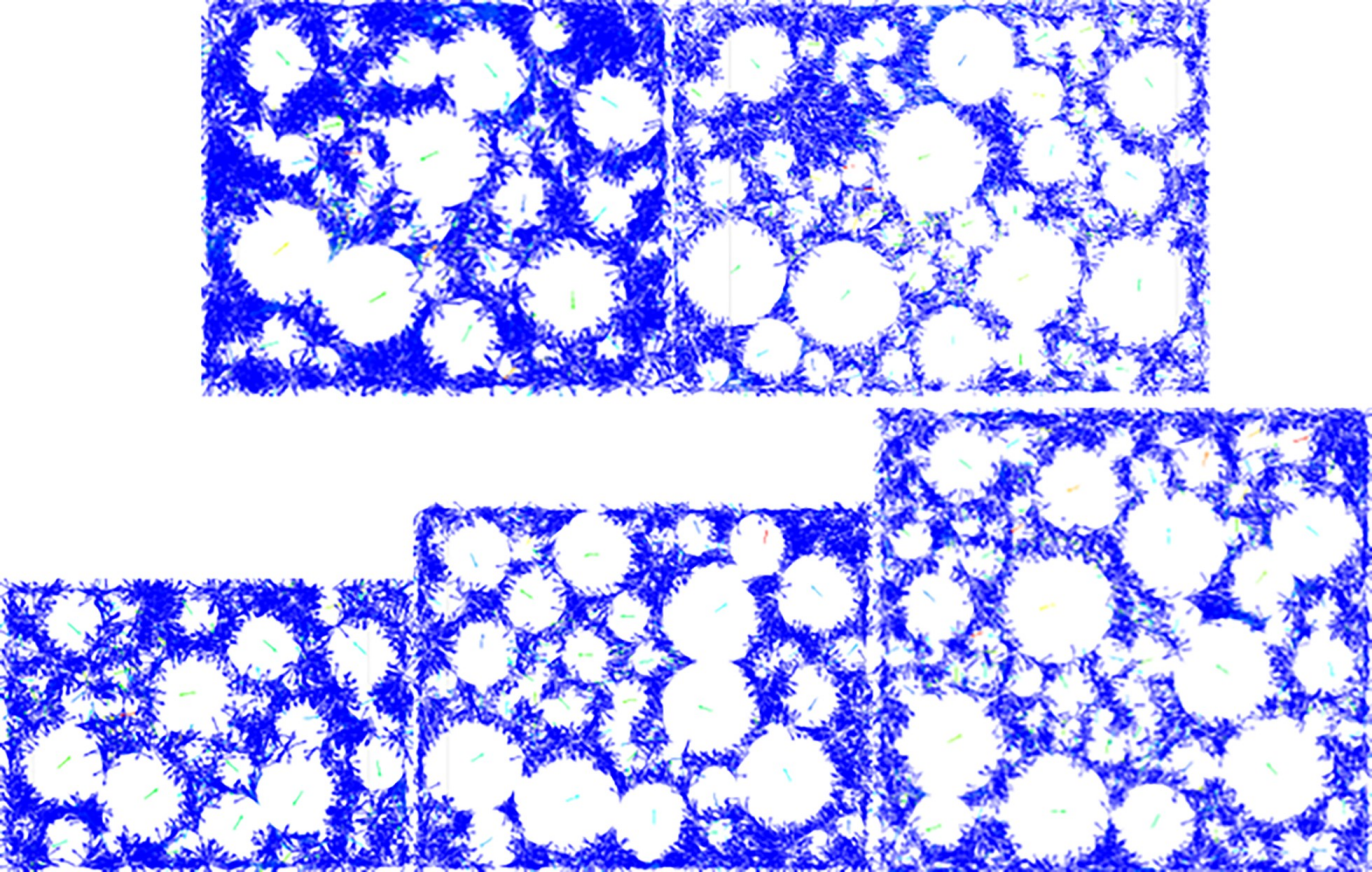
Displacement vector field of different size specimens under uniaxial compression test: (a)180 mm×160 mm, (b) 220 mm×160 mm, (c) 200 mm×140 mm, (d) 200 mm×160 mm, (e) 200 mm×180 mm.

**Fig 8:**
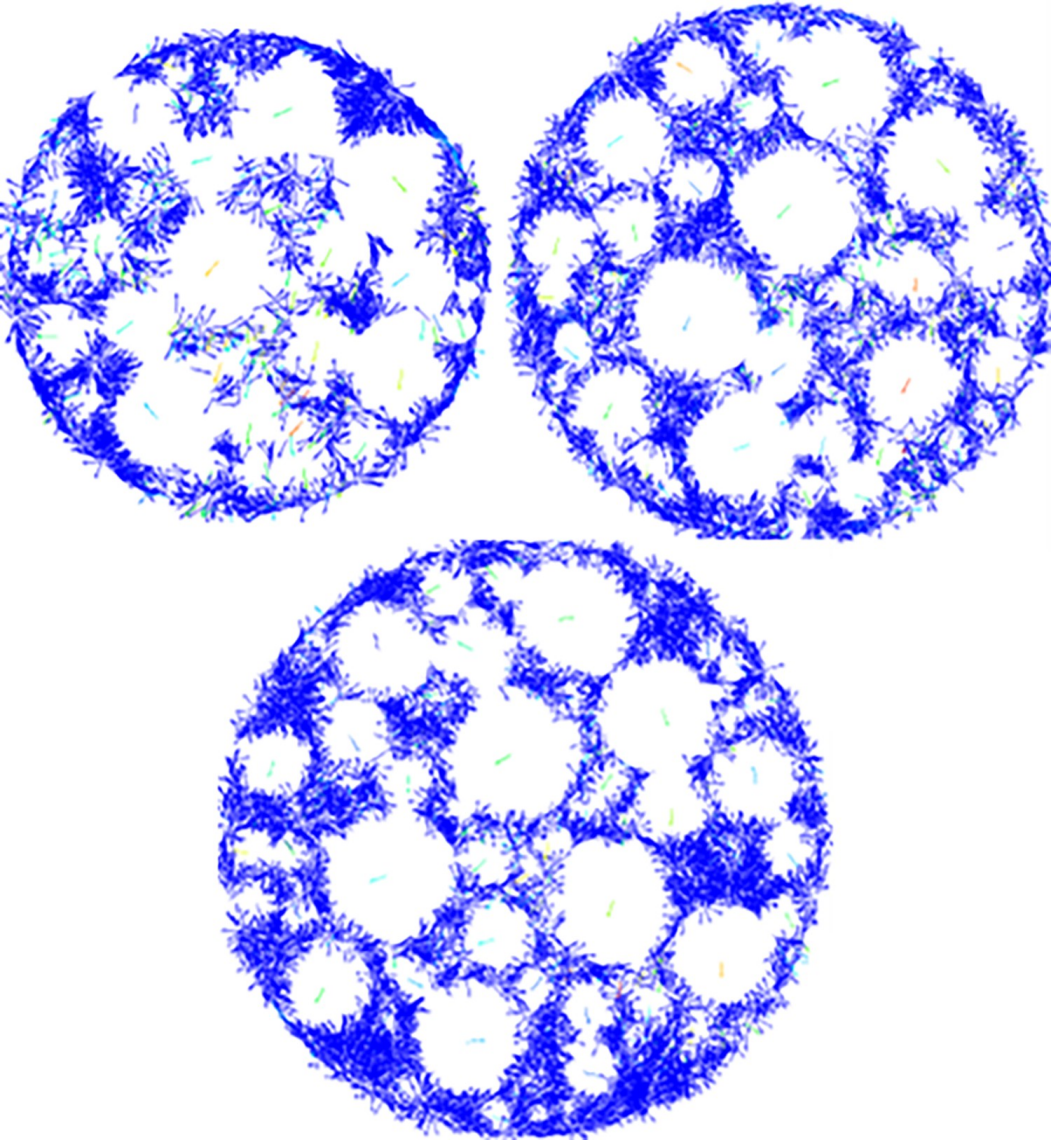
Displacement vector field of different size specimens under split test: (a)180 mm×160 mm, (b) 200 mm×160 mm, (c) 220 mm×160 mm.

Discrete element software offers certain advantages in observing micromechanisms. The numerical model constructed in this paper can also clearly depict the movement of particles inside the specimen during the loading process. To further analyze the impact of the LSAM-50 mixture specimen size on mechanical strength, this section uses the displacement vector diagrams of Grade JP50-1 specimens during the numerical experiment to reveal the micromechanisms under different specimen sizes. The compressive strength and splitting strength displacement vector fields of Grade JP50-1 LSAM-50 mixture under different specimen sizes are shown in Figs 7 and 8.

According to Fig 7, when the height of the LSAM-50 specimen is less than 160 mm, the displacement trend of aggregates within the specimen is quite pronounced. Under the influence of the loading plate, the aggregates at the top and bottom of the specimen undergo vertical displacement due to pressure. When the specimen height is less than 160 mm, only a few of these vertical displacement vectors cancel each other out, leading to a tendency for horizontal displacement under the constraint of the loading and base plates. As the specimen height exceeds 160 mm, most of the vertical displacement vectors are effectively neutralized. With further increases in specimen height, the tendency for particles to move horizontally gradually weakens, indicating a decreasing influence of the loading plate as the LSAM-50 specimen fails.

Fig 8 shows that as the diameter of the specimen increases, the displacement trend of the aggregates within the LSAM-50 specimen becomes more stable. When the specimen diameter is less than 200 mm, the lateral displacement vectors of the particles are larger due to the constraints of the loading plate and base plate. Once the specimen diameter exceeds 200 mm, lateral displacement vectors are concentrated within the specimen and gradually dissipate along the diameter.

In summary, the proposed size for LSAM-50 numerical experiment specimens is Φ200 mm × h160 mm.

## 6. Verification of reliability for the LSAM-50 mixture numerical model

**Fig 9:**
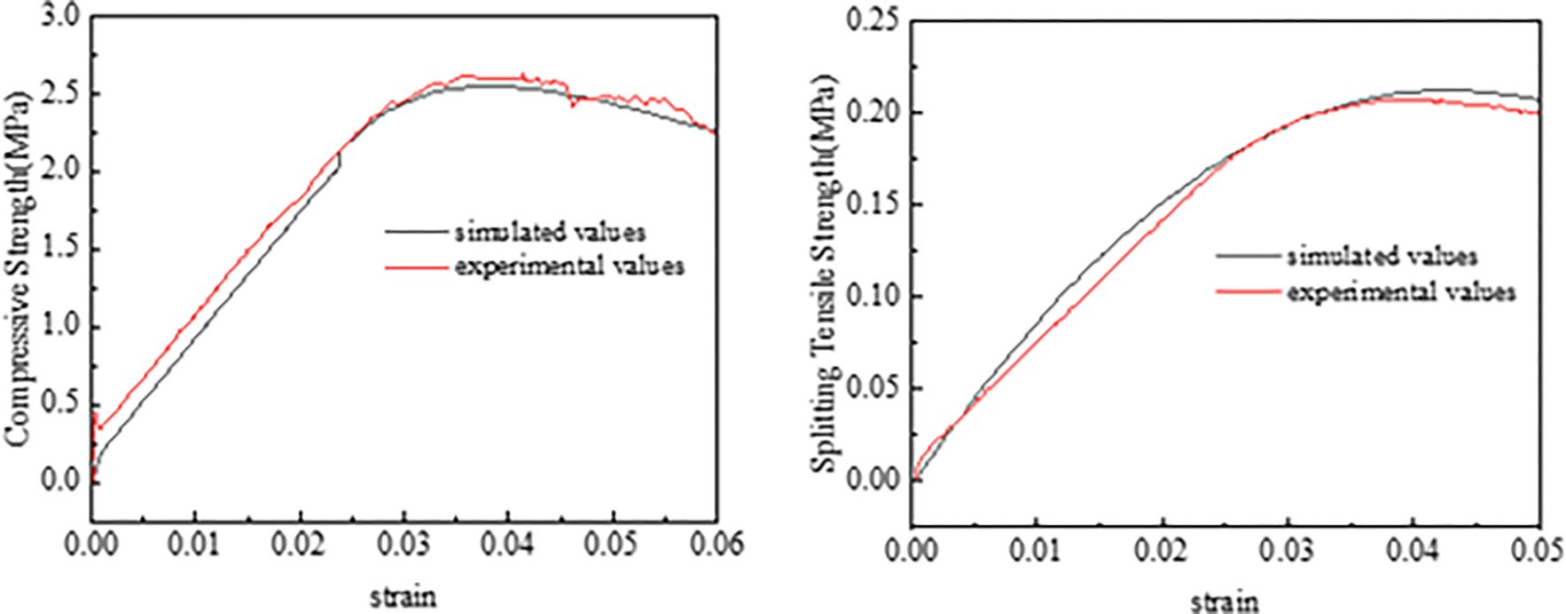
The stress-strain curve of JP50-1 graded LSAM-50 mixture: (a) compressive strength stress-strain curve, (b) splitting strength stress-strain curve.

Utilizing the model parameters obtained from Table 15, uniaxial compression numerical tests and splitting numerical tests were conducted. The simulated and actual measured values for different gradations of LSAM-50 mixture are presented in Table 16. The stress-strain curves during the uniaxial compression test and splitting test for Grade JP50-1 are shown in Fig 9.

**Table 16:** Simulation value and error of LSAM-50 with different grades.

Gradation	JP50-1	JP50-2	JP50-3	JP50-4
Compressive Strength Simulation Value (MPa)	2.66	2.77	2.82	2.88
Compressive Strength Experimental Value (MPa)	2.59	2.68	2.72	2.76
Error in Compressive Strength (|%|)	2.70	3.36	3.68	4.35
Splitting Tensile Strength Simulation Value (MPa)	0.207	0.21	0.216	0.219
Splitting Tensile Strength Experimental Value (MPa)	0.214	0.216	0.219	0.222
Error in Splitting Tensile Strength (|%|)	3.27	2.78	1.37	1.35

As indicated in Table 16 and Fig 9, the error between the simulated and experimental values of the splitting tensile strength of the LSAM-50 mixture is within 3%, and the error for compressive strength is within 5%. The stress-strain curves corresponding to the simulated values of compressive and splitting tensile strengths closely match the actual measured stress-strain curves. This alignment substantiates the reliability of the numerical testing method for the LSAM-50 mixture.

## 7. Conclusion

(1) The particles for the LSAM-50 numerical specimens were generated by means of a stepwise expansion method. Numerical test models and simulation processes for uniaxial compression and splitting tests of LSAM-50 mixtures were established. The sensitivity of model parameters was analyzed, and the parameters were calibrated.

(2) The conditions for LSAM-50 numerical tests were investigated. It was found that a computational time step of 10⁻^3^ s/step and loading rates of 2 mm/min (for uniaxial compression numerical tests) and 50 mm/min (for splitting numerical tests) provided a good balance between computation speed and accuracy in LSAM-50 numerical tests.

(3) Considering the influence of LSAM-50 specimen size on mechanical strength and displacement vector fields, it was observed that when the size exceeded 200×160mm, changes in mechanical strength were not significant, indicating the elimination of the influence of size effect. Although increasing the specimen size could enhance the accuracy of laboratory tests, it also increased the complexity of these tests. A reasonable specimen size of Φ200mm×h160mm for LSAM-50 is proposed.

(4) The reliability of LSAM-50 numerical tests was verified through laboratory experiments. The errors in uniaxial compression and splitting numerical tests of LSAM-50 compared with laboratory tests were ≤5% and ≤3%, respectively.

## Supporting information

S1 File This file includes all the test data of the asphalt binders and asphalt mixtures. (PDF)
