# Supplementary material for: Numerical testing method and mechanical property evaluation of large particle size asphalt mixture
Source: PLoS One. 2025 Jan 15;20(1):e0316191. doi: 10.1371/journal.pone.0316191 (PMC11734954; doi:10.1371/journal.pone.0316191)
Supplement: S1 File — (PDF) [file pone.0316191.s001.pdf]

S1 File. Data. This file includes all the test data of the asphalt binders and asphalt mixtures.

Table 1. Asphalt technical properties

Items		Tested results	Standard value
Penetration (25 °C, 100 g, 5 s) (0.1 mm)		68	60～80
Ductility (5 cm/min, 15 °C) (cm)		＞100	⩾100
Softening point(°C)		47.3	⩾46
Penetration index (PI)		-0.315	-1.5～+1.0
Flashpoint(°C)		290	⩾260
Solubility in trichloroethylene (%)		99.6	⩾99.5
Rolling Thin Film Oven Test (163°C,5h)	Quality loss (%)	0.03	⩽±0.8
	Ductility (10°C) (cm)	7	⩾6
	Penetration ratio (25°C)(%)	63	⩾61

Table 2. Technical properties of aggregate

Aggregate	Items	Tested results	Standard value
Coarse aggregate	Crushed value (%)	19.3	⩽26
	Los Angeles Abrasion Value / %	19.8	⩽28
	Flaky particle content (%)	8.1	⩽15
	Apparent relative density(g/cm^3)	2.781	⩾2.6
Fine aggregate	Methylene blue value (g/kg)	3.7	⩽25
	Sturdiness (%)	6.9	⩽12
	Apparent relative density(g/cm^3)	2.719	⩾2.5

Table 3. Aggregate gradation of LSAM-50

Gradation	Percentage by mass passing through the sieve sizes (mm)											
	63	53	37.5	19	9.5	4.75	2.36	1.18	0.6	0.3	0.15	0.075
JP50-1	100	100	67	45	35	30	25	17.5	13	9.5	6.5	3
JP50-2	100	100	65	45	35	30	25	17.5	13	9.5	6.5	3
JP50-3	100	100	67	49	35	30	25	17.5	13	9.5	6.5	3
JP50-4	100	100	70	55	35	30	25	17.5	13	9.5	6.5	3

Table 4. Volumetric Parameters and Road Performance of LSAM-50 Mixture

Indicator	Bulk Density /（g/cm^3）	Void Ratio/ %	Aggregate Void Ratio /%	Asphalt Saturation/ %	Dynamic Stability /（time/mm）	Bending Strain（με）	SCB Strength/MPa	Residual SCB Strength/ %
value	2.548	3.7	7.6	50.5	14021	3357	10.71	90.4

Table 5. Initial model parameters

Parameter Type	*f*	k_n (GPa)	k_s (GPa)	*v*	*krat*	*pb_ten* (MPa)	*pb_k*$_n$ (GPa)	*pb_k* (GPa)
Parameter Value	0.38	33	13.0	0.3	2.60	0.2	33	0.1

Table 6. The simulated values and errors of different gradations under the initial model parameters

Gradation	JP50-1	JP50-2	JP50-3	JP50-4

Compressive Strength（MPa）	2.73	2.85	2.89	2.95
Splitting Tensile Strength（MPa）	0.230	0.232	0.236	0.239
Error in Compressive Strength（|%|）	5.41	6.34	6.25	6.88
Error in Splitting Tensile Strength（|%|）	7.48	7.41	7.76	7.66

Table 7. The simulated values and errors of different gradations under the adjusted model parameters

Gradation	JP50-1	JP50-2	JP50-3	JP50-4
Compressive Strength（MPa）	2.66	2.77	2.82	2.88
Splitting Tensile Strength（MPa）	0.207	0.21	0.216	0.219
Error in Compressive Strength（|%|）	2.70	3.36	3.68	4.35
Error in Splitting Tensile Strength（|%|）	3.27	2.78	1.37	1.35

Table 8. Final model parameters

Parameter Type	*f*	k_n (GPa)	k_s (GPa)	*v*	*krat*	*pb_ten* (MPa)	pb_k_n (GPa)	*k* (GPa)
Parameter Value	0.35	33	13.0	0.3	2.60	0.18	30	0.5

Table 9. Effect on the mechanical strength of the specimen size LSAM-50 Mixture

Specimen Size/mm	Test Indicators	Mechanical Strength of LSAM-50 Mixture for the Following Gradations / MPa			
		JP50-1	JP50-2	JP50-3	JP50-4
180×160	Compressive Strength	2.82	2.91	3.07	3.13
	Splitting Tensile Strength	0.218	0.225	0.229	0.231
200×140	Compressive Strength	2.75	2.89	3.02	3.07
	Splitting Tensile Strength	0.237	0.242	0.248	0.251
200×160	Compressive Strength	2.66	2.77	2.82	2.88
	Splitting Tensile Strength	0.207	0.211	0.217	0.219
200×180	Compressive Strength	2.61	2.68	2.73	2.79
	Splitting Tensile Strength	0.184	0.188	0.193	0.195
220×160	Compressive Strength	2.57	2.69	2.73	2.80
	Splitting Tensile Strength	0.189	0.194	0.201	0.205

Table 10. Simulation value and error of LSAM-50 with different grades

Gradation	JP50-1	JP50-2	JP50-3	JP50-4

Compressive Strength Simulation Value（MPa）	2.66	2.77	2.82	2.88
Compressive Strength Experimental Value（MPa）	2.59	2.68	2.72	2.76
Error in Compressive Strength（|%|）	2.70	3.36	3.68	4.35
Splitting Tensile Strength Simulation Value（MPa）	0.207	0.21	0.216	0.219
Splitting Tensile Strength Experimental Value（MPa）	0.214	0.216	0.219	0.222
Error in Splitting Tensile Strength（|%|）	3.27	2.78	1.37	1.35
